# Redox-Dependent Copper Ion Modulation of Amyloid-β (1-42) Aggregation In Vitro

**DOI:** 10.3390/biom10060924

**Published:** 2020-06-18

**Authors:** Nima Sasanian, David Bernson, Istvan Horvath, Pernilla Wittung-Stafshede, Elin K. Esbjörner

**Affiliations:** Department of Biology and Biological Engineering, Chalmers University of Technology, 412 96 Gothenburg, Sweden; sasanian@chalmers.se (N.S.); david.bernson@chalmers.se (D.B.); istvanh@chalmers.se (I.H.); pernilla.wittung@chalmers.se (P.W.-S.)

**Keywords:** amyloid, amyloid-β, copper, aggregation, kinetics, inhibition, Alzheimer’s disease

## Abstract

Plaque deposits composed of amyloid-β (Aβ) fibrils are pathological hallmarks of Alzheimer’s disease (AD). Although copper ion dyshomeostasis is apparent in AD brains and copper ions are found co-deposited with Aβ peptides in patients’ plaques, the molecular effects of copper ion interactions and redox-state dependence on Aβ aggregation remain elusive. By combining biophysical and theoretical approaches, we here show that Cu^2+^ (oxidized) and Cu^+^ (reduced) ions have opposite effects on the assembly kinetics of recombinant Aβ(1-42) into amyloid fibrils in vitro. Cu^2+^ inhibits both the unseeded and seeded aggregation of Aβ(1-42) at pH 8.0. Using mathematical models to fit the kinetic data, we find that Cu^2+^ prevents fibril elongation. The Cu^2+^-mediated inhibition of Aβ aggregation shows the largest effect around pH 6.0 but is lost at pH 5.0, which corresponds to the pH in lysosomes. In contrast to Cu^2+^, Cu^+^ ion binding mildly catalyzes the Aβ(1-42) aggregation via a mechanism that accelerates primary nucleation, possibly via the formation of Cu^+^-bridged Aβ(1-42) dimers. Taken together, our study emphasizes redox-dependent copper ion effects on Aβ(1-42) aggregation and thereby provides further knowledge of putative copper-dependent mechanisms resulting in AD.

## 1. Introduction

Alzheimer’s disease (AD) is an incurable and fatal neurodegenerative condition resulting from the progressive death of neurons in the brain and the concomitant loss of cognitive functions, and which is associated with protein aggregation and deposition [1]. AD is the most common form of adult dementia, mainly afflicting people over the age of 65 [2]. The prevalence of the disease is expected to rise worldwide due to population ageing, and unless there are new disease-modifying treatments, a projected 130 million people may be affected by 2050 [3]. 

**Figure 1:**
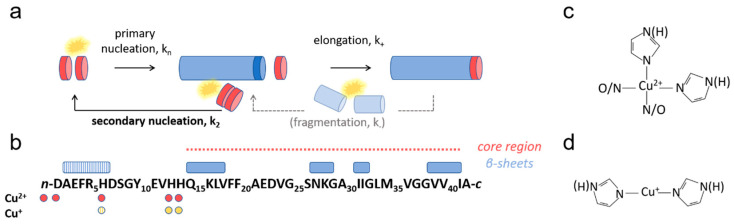
Amyloid formation and copper coordination by amyloid-β (Aβ)(1-42). (**a**) Schematic illustration of the reaction steps involved in nucleated amyloid fibril formation, including primary nucleation, elongation, secondary nucleation, and fragmentation. The kinetics of the aggregation of Aβ(1-42) alone is dominated by the generation of new aggregates through secondary nucleation (bold text), whereas fragmentation (shadowed) plays no significant role. (**b**) Aβ(1-42) primary structure with proposed amyloid fibril core β-sheets, according to recent high-resolution structural models indicated by blue boxes [73,74]. Note that one model reports β-sheet formation also at the N-terminus, albeit without participation in the formation of the core. Coordination sites for Cu^2+^ and Cu^+^ are indicated by red and yellow dots, respectively. (**c**,**d**) Consensus coordination mode of (**c**) Cu^2+^ and (**d**) Cu^+^, with respective ligation partners [65].

AD is pathologically, genetically, and biochemically strongly linked to amyloid formation and the deposition of amyloid-β (Aβ) peptides into extracellular senile plaques [1,4,5]. Aβ peptides are generated by the proteolytic cleavage of the so-called amyloid precursor protein (APP) by β- and γ-secretases [6]. This occurs in the acidic environment of endosomes [7], where Aβ can also aggregate [8] and accumulate during early neuronal dysfunction and prior to the deposition of extracellular plaques [9]. Aβ peptides can differ in size. Even though the 40 amino acid Aβ(1-40) variant is most abundant, the two residues longer Aβ(1-42) variant is more aggregation-prone [10] and also the main protein constituent of extracellular amyloid plaques [11]. Probing Aβ(1-42) self-assembly mechanisms and the influence of biological as well as pharmacological extrinsic aggregation modifiers is therefore important. Recent progress in the development of models that mechanistically describe how amyloid fibrils are formed [12,13] has enabled the determination of rate constants for different microscopic reaction steps (Figure 1a) through the analysis of amyloid kinetics data. In the case of Aβ(1-42), this analysis has revealed that secondary nucleation is the dominant mechanism of new aggregate formation [14]. The same methods have also been used to rationalize the inhibitory effects of chaperones [15] and antibodies [16], and the catalytic effect of small molecules [17] and lipids [18].

Metal ions and imbalances in metal ion homeostasis are considered to play pivotal roles in neurodegeneration [19,20,21], contributing to oxidative stress responses [22,23] as well as directly to protein aggregation [24,25,26,27]. Along this line, metal ions—including iron, zinc, and copper (Cu)—are found to co-deposit with Aβ fibrils in the core of plaques [28,29,30], and interactions between Aβ and redox-active metal ions (e.g., Fe and Cu) can drive the production of hydrogen peroxide [31,32,33], superoxide anions [34], and hydroxyl radicals [35], thus potentially contributing directly to the oxidative stress that is observed in AD [36]. Based on this type of data, metal chelators have been proposed as future AD therapeutics [37], and favorable effects on Aβ accumulation and toxicity both in vitro and in vivo have been reported [38]. However, the literature on how metal ions affect Aβ peptide aggregation is inconclusive in many respects, with reports on both enhancing and inhibitory effects.

Dysregulated Cu homeostasis has been associated with AD [19,39,40,41,42] and is manifested as elevated Cu levels in plasma [19,41] but diminished levels of Cu in the brain, especially in the amygdala and hippocampal regions [40]. The concentrations of total Cu in amyloid plaques (400 μM) and the synaptic cleft (15 μM) are higher than the extracellular concentration in the brain (0.2–1.7 μM) [21]; the former may contribute to the reported imbalances. The redox ability of Cu enables it to cycle between Cu^+^ and Cu^2+^ states, underlying its function in many enzymes. The Cu^2+^ form is predominant under oxidizing conditions which also prevail in many locations where Aβ is abundant, such as the extracellular space and the oxidizing and low pH environment of endosomes and lysosomes [43]. In the context of AD, Cu^2+^ is therefore the most frequently studied redox form. Cu^+^, which is mainly bound to proteins in the cytosol where the environment is highly reducing, has been less explored, but the high reduction potential found for Cu when bound to Aβ suggests that Cu^+^:Aβ may be present at oxidative stress and/or pathological conditions. Probing the effects of both the redox states of Cu on Aβ aggregation is therefore important. 

There are several biophysical studies on the effect of Cu^2+^ on Aβ aggregation, but the results are divergent. The majority of papers suggest that Cu^2+^ inhibits Aβ aggregation [44,45,46,47,48,49,50], a property shared with, for example, Zn^2+^ [51]. However, accelerating effects have also been suggested [52,53,54], for example under mildly acidic conditions [24]. Cu^2+^ has also been reported to selectively induce the formation of cytotoxic Aβ(1-42) oligomers under conditions where Zn^2+^ did not [55], thereby putatively contributing specifically to toxicity. In addition, Cu^2+^ have been reported to modulate Aβ integrity, for example by inducing the aggregation-inhibitory dityrosine dimerization in the presence of H_2_O_2_ or ascorbate [52], or by mediating hydrolytic cleavage in presence of large excess of antioxidants [56], emphasizing that this metal has multiple effects. The role of Cu^+^ in Aβ(1-42) aggregation has been less studied, but one report suggested, based on transmission electron microscopy, that both Cu^+^ and Cu^2+^ could prevent Aβ fibril formation [57]. 

Aβ peptides coordinate both Cu^2+^ and Cu^+^ ions via N-terminal residues (Figure 1a), but there is significant disparity in their binding affinities and exact coordination geometries. For Cu^2+^, dissociation constants are reportedly in the picomolar to low nanomolar range [58], and both 1:1 and 2:1 binding stoichiometries have been reported, albeit with significantly weaker binding of the second Cu^2+^ ion [59,60,61,62]. Aβ binds Cu^2+^ primarily via 3N1O coordination, resulting in the formation of an N-terminal loop via the engagement of the N-terminus, the carboxyl group on Asp1, and two imidazole nitrogens (on His6, His13, or His 14) or Asp1 and all three histidines (Figure 1b,c) [60,63,64,65,66]. The binding affinity [67], coordination geometry [64,66,68,69], and stoichiometry [70] of Cu^2+^ is sensitive to pH. A loss of coordination or weak affinity at pH 5.0 has been reported [67], as well as the successive transition towards 4N coordination above pH 8.0 [66]. Probing how Aβ aggregation proceeds as a function of pH is therefore important, particularly in light of the pathological aggregation of Aβ(1-42) in endolysosomes [8,9] where metals, including Cu, are conspicuously abundant [71]. Cu^2+^ can bind monomeric, oligomeric, and fibrillar Aβ states with comparable affinity [63,64], and it was reported that the level of Cu^2+^ bound to Aβ(1-40) did not change during aggregation [63]. This is consistent with the fact that residues 1-14 of Aβ(1-42) (to which Cu coordinates) remain flexible and are not incorporated in the amyloid fibril core (Figure 1b). Cu^+^ binds Aβ in a bidentate linear 2N coordination engaging two N-imidazoles (Figure 1d), most likely His13 and His14 [65]. Thus, Cu^+^ binding can occur without loop formation, rendering the N-terminus more flexible compared to when binding Cu^2+^. The affinity of Cu^+^ is reported to be in the femtomolar range, suggesting that Cu^+^ binds tighter than Cu^2+^ to Aβ [65,72]. Notably, there are no reports on the interactions of Cu^+^ with Aβ oligomers or fibrils. 

In this study, we revisit the question of how Cu influences Aβ(1-42) amyloid formation, using Thioflavin-T assays with monomeric recombinant peptides as the starting material [75], a procedure that we [18] and others [14] have shown to result in highly reproducible aggregation kinetics. Moreover, by taking advantage of recent theoretical developments to model protein aggregation kinetics [14], enabling detailed molecular and mechanistic understanding of amyloid formation catalysis and inhibition as mentioned above, this study reveals new quantitative information on how Cu^2+^ and Cu^+^ affect discrete microscopic events during Aβ(1-42) self-assembly, resulting in different macroscopic effects on aggregation by the two forms of Cu. We also explore the pH-dependent effects of Cu^2+^ ions on Aβ(1-42) aggregation, addressing the pathological consequences of alterations in pH during endolysosomal processing, which may relate to the earliest Aβ aggregation events [73,74]. Our main finding is that Cu^2+^ and Cu^+^ have opposite effects on Aβ(1-42) aggregation. Cu^2+^ primarily interferes with fibril elongation, whereas Cu^+^ instead catalyzes primary nucleation. We discuss the results in relation to the Cu coordination geometries and the putative effects of Cu on Aβ-mediated neurotoxicity in the context of AD. 

## 2. Materials and Methods 

### 2.1. Protein Expression and Purification

Recombinant Aβ(1-42) was expressed as a fusion protein with the solubility tag NT* and purified as described in [76,77], but with some modification. The plasmid encoding for NT-Aβ(1-42) (a kind gift from Dr. Henrik Biverstål, Karolinska Institute) was transfected into *E. coli* (BL21) cells and expressed overnight using 0.5 mM IPTG induction at OD_600_~0.7. Bacterial cells were harvested by centrifugation and lysed in 20 mM Tris-HCl, 8 M urea, pH 8.0, by sonication. The sonicated lysate was filtered through a 0.22 µM syringe filter (VWR, Radnor, US) and loaded onto a HisPrep FF 16/10 column (GE Healthcare, Chicago, US) equilibrated with 20 mM Tris-HCl, 8 M urea, pH 8.0. The NT-Aβ(1-42) was eluted with 300 mM imidazole in the same buffer and then dialyzed against 5 L 20 mM sodium phosphate at 4 °C, with 1.5 mM 1,4-dithiothreitol (DTT) and 0.5 mM EDTA, pH 8.0. After 2 hrs of dialysis, 1:20 molar equivalents of tobacco etch virus (TEV) protease (produced as previously described [78]) was added to the NT-Aβ(1-42), followed by quiescent incubation over night at 4 °C. Following the TEV digestion, the solution was injected onto a HiLoad 16/600 Superdex 30 pg (GE Healthcare, Chicago, US) size exclusion column equilibrated with 20 mM sodium phosphate, pH 8.0, and monomeric Aβ(1-42) was isolated as a single peak. The monomeric peptides were immediately aliquoted, freeze dried, and stored at −20 °C until further use. 

### 2.2. Aβ(1-42) Aggregation Assays

The Aβ(1-42) aggregation was monitored by thioflavin-T (ThT) fluorescence. Prior to each kinetic experiment, lyophilized Aβ(1-42) was dissolved in 6 M guanidine hydrochloride (GuHCl), followed by incubation on ice for 20 min. The monomeric Aβ(1-42) solution was purified by size-exclusion chromatography using a 10/300 Superdex 75 (GE Healthcare, Chicago, IL, US) column and equilibrated with 20 mM sodium phosphate, pH 8.0, to minimize isoelectric precipitation and reduce the aggregation rate so that the samples could be transferred to microtiter plates without significant aggregation occurring, following a common experimental protocol [14,79,80]. The Aβ(1-42) concentration was determined from the integrated peak area using an extinction coefficient of ε_280_ = 1280 M^−1^cm^−1^. The monomeric Aβ(1-42) solution was immediately transferred to ice to prevent any aggregation before starting the experiments. The Aβ(1-42) monomers were diluted in phosphate buffers or citrate-phosphate buffers to obtain a specific solution pH, as indicated in the text and figure legends (see Table S1 for detailed buffer compositions). 5 µM thioflavin-T (ThT, Sigma, St. Louis, MO, US) (three times recrystallized in tetrahydrofuran (THF) to remove impurities) was added from a 0.5 mM stock solution. The Aβ(1-42) solutions were supplemented with CuCl_2_ (to obtain Cu^2+^) or CuCl_2_ pre-mixed with a 5x molar excess of freshly prepared 1,4-dithiothreitol (DTT, Sigma, St. Louis, MO, US) to generate Cu^+^, according to a previously established protocol [81,82,83]. The peptide solutions were thereafter added to the wells of 96-well black half-area, low binding, transparent bottom microtiter plates (Corning, #3881, Corning, NY, US). The total volume in each well was always 70 µL. The plates were sealed with adhesive film (BIO-RAD, Hercules, US) to prevent sample evaporation and the ThT emission was measured from the bottom of the plate using a Fluostar Optima or Fluostar Omega plate reader (BMG Labtech, Ortenberg, Germany ) with a 440 ± 10 nm bandpass filter for excitation, and a 490 ± 10 nm bandpass filter for emission. All measurements were performed in triplicate (n = 3) and under strictly quiescent conditions (no shaking) at 37 °C and were repeated at least 3 times. 

### 2.3. Seeds Preparation

Fibril seeds for seeded aggregation experiments were formed by setting up the above-described kinetic experiment with 2.6 µM Aβ(1-42) in 20 mM sodium phosphate buffer, pH 8.0, and 5 µM ThT. All seeds were prepared in the absence of Cu. The seeds were collected from the wells after the completion of the experiment and transferred into low-binding 1.5 mL tubes and then added to samples based on the monomer-equivalent concentration. 

### 2.4. Analysis and Fitting of ThT Kinetic Curves

Kinetic data acquired in the presence of increasing amounts of Cu^+^ and Cu^2+^ were analyzed based on the previously described integrated rate laws for Aβ(1-42) amyloid formation and using a model including secondary nucleation [14,84]. The theoretical curves of the kinetic evolution of aggregates were globally fitted to the experimental ThT data (see Section 2.2) using the AmyloFit web interface [85] to obtain quantitative estimates of the rate constants. For Cu^2+^, we used a seeded model to determine individually the rate constants for primary nucleation (k_n_), elongation (k_+_), and secondary nucleation (k_2_), keeping one parameter at a time variable (i.e., dependent on the Cu^2+^ concentration) and the others as global constants (i.e., independent of the Cu^2+^ concentration). All the fits were conducted using a 10-basin hop algorithm with errors and using normalized ThT kinetics data, as described by Meisl et al. [85]. For Cu^+^, we used a non-seeded model to determine the convoluted rate constants k_+_k_n_ and k_+_k_2_.

### 2.5. Dot-Blot Assay

Dot-blot assays were performed using the anti-amyloid fibril antibody LOC (Millipore, Burlington, MA, US, [86]), specific for amyloid fibrils. 20 µL of each sample containing Aβ(1-42) with or without Cu^2+^ or Cu^+^ was taken directly from the aggregation reactions and added to a 96-well dot-blot hybridization manifold (Scie-plas Ltd., Cambridge, UK). Vacuum pressure was applied to deposit the samples onto an LF-PVDF membrane (Trans-Blot, BIO-RAD, Hercules, CA, US). The membrane was dried and blocked with 30 mL of 5% skimmed milk in phosphate buffer saline with Tween-20 (PBS-T) and incubated for 1 h at room temperature. The membrane was then incubated for 30 min at room temperature with 30 mL of a primary antibody (1:100,000) dissolved in BSA/PBS-T. After three washes (5 min each time) with PBS-T, the membrane was incubated with a 1:20,000 dilution HRP-conjugated goat anti-rabbit secondary antibody (Sigma, St. Louis, MO, US). Thereafter, the membrane was washed three times with PBS-T (15 min × 1, 5 min × 2). The resulting dots were developed using an enhanced chemiluminescence solution (ECL, GE healthcare, Chicago, IL, US) together with the chemiluminescence detection system of a ChemiDoc^TM^ gel scanner (BIO-RAD, Hercules, CA, US). The densiometric analysis was performed using the instrument’s built-in software and the region-of-interest function.

### 2.6. Transmission Electron Microscopy (TEM)

TEM images were taken to examine the morphological appearance of Aβ(1-42) amyloid aggregates in the absence and presence of Cu. 10 µL of the sample taken at the end-point of the kinetic aggregation experiment was deposited onto glow-charged, formvar-coated, carbon-stabilized copper EM grids (Agar scientific, S138, Essex, UK) and allowed to settle for 10 min. The grids were thereafter stained with aqueous phosphotungstate (1% PTA), blotted with filter paper (Whatman, Grade 40, GE Healthcare, Chicago, IL, US), and allowed to dry before imaging [87]. High-contrast images were obtained with a TALOS L120C TEM (Thermo Fisher Scientific, Waltham, MA, US) at an accelerating voltage of 120 kV using a 4 × 4 k CMOS Ceta camera (Thermo Fisher Scientific, Waltham, MA, US). The magnification was set to 17,500x–300,000x.

## 3. Results and Discussion

### 3.1. Cu^2+^ Inhibits Aβ(1-42) Amyloid Formation

**Figure 2:**
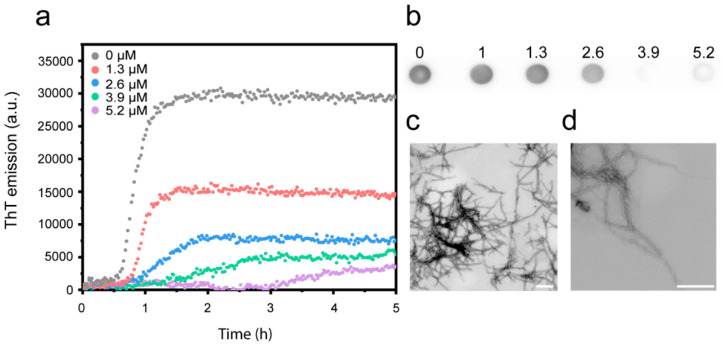
Aβ(1-42) amyloid formation at pH 8.0 in the presence of Cu^2+^. (**a**) Amyloid formation kinetics of 2.6 µM Aβ(1-42) measured by thioflavin-T (ThT) (5 µM) fluorescence in the presence of indicated concentrations of CuCl_2_. Data are shown as representative curves; the full set of replicates (n = 3) are shown in Figure S2a. (**b**) Dot blot showing LOC-positive Aβ(1-42) species at the endpoint of the kinetic experiments shown in (**a**). The full, uncropped image of the dot-blot membrane is shown in Figure S4. (**c**,**d**) TEM images of 2.6 µM Aβ(1-42) amyloid fibrils formed in the presence of (**c**) 1.3 µM and (**d**) 5.2 µM Cu^2+^. The scale bars represent 200 nm.

To explore the effect of Cu^2+^ ions on the aggregation of Aβ(1-42) into fibrils, we used Thioflavin-T (ThT) fluorescence to measure the amyloid formation kinetics. ThT binds to the regular β-sheet structure of amyloid fibrils (but does not associate with unstructured oligomers), and this increases dramatically its fluorescence quantum yield [88]. At stoichiometric amounts, ThT has been shown to have a negligible effect on the aggregation kinetics [14], and it is therefore a good in situ reporter of amyloid fibril formation. Recombinant Aβ(1-42) was monomerized by size exclusion chromatography (SEC) (Figure S1) immediately prior to each experiment to avoid the presence of pre-formed aggregates [75,85]. The purified peptides were allowed to aggregate under quiescent conditions in the absence or presence of CuCl_2_, such that the experiment probed the Cu^2+^:Aβ ratios of 0.5:1, 1:1, 1.5:1 and 2:1. The results (Figure 2a, Figure S2a) show that presence of Cu^2+^ inhibits the aggregation of Aβ(1-42) in a dose-dependent manner, resulting in both longer aggregation half-times and reduced maximum levels of ThT emission. We confirmed that the inhibitory effect is specific to Cu^2+^ by demonstrating that another divalent metal ion (Mg^2+^) had no, or a very marginal effect, even when added in 30x molar excess (Figure S3). 

Using the conformation-sensitive anti-amyloid fibril antibody LOC [86] to detect fibrils, the dot-blot analysis reveals that the fibril yield decreases with increasing Cu^2+^ concentration (Figure 2b, Figure S4). A densiometric analysis suggests a ~90% reduction in fibrils at the highest Cu^2+^ concentration (Figure S5a). This confirms that the decrease in ThT intensity reflects inhibition and is not merely an effect of Cu^2+^-mediated fluorescence quenching [89]. Control experiments showed, however, that some Cu-mediated quenching also occurs; 34% of the ThT emission in a sample with pre-formed Aβ(1-42) fibrils was lost at an Cu^2+^:Aβ ratio of 1:1, but the effect was reversible upon the addition of EDTA (Figure S6). This is consistent with the result that the ThT emission at a 1:1 ratio decreased more than expected from the reduction in the LOC-detected fibril yield (Figure S5a). 

Our findings are in good qualitative agreement with several other biophysical studies [44,47,48,49,50], and thus add to the growing consensus that Cu^2+^ ions inhibit Aβ(1-42) aggregation. Whilst a few studies report that Cu^2+^ ions induce the formation of amorphous aggregates of Aβ(1-42) [48,90], the TEM analysis revealed the formation of mature amyloid fibrils at both sub- and super-stoichiometric Cu^2+^ concentrations (Figure 2c,d) in our case.

### 3.2. pH and Salt Dependence of the Cu^2+^-Mediated Inhibition of Aβ(1-42) Amyloid Formation

**Figure 3:**
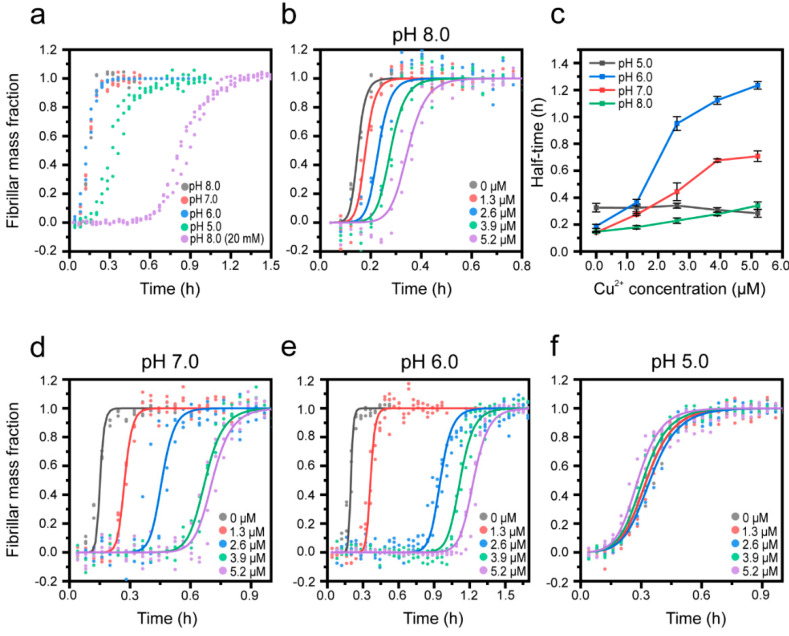
Cu^2+^ inhibition of Aβ(1-42) amyloid formation at different pHs. (**a**) Normalized kinetic profiles of 2.6 µM Aβ(1-42) at various pHs. (**b**) Normalized kinetic profiles of 2.6 µM Aβ(1-42) in a 200 mM sodium phosphate buffer, pH 8.0, and in the presence of Cu^2+^. (**c**) Half-times of the aggregation of 2.6 µM Aβ(1-42) at various pHs in the presence of increasing concentrations of Cu^2+^. (**d**–**f**) Normalized kinetic profiles of 2.6 µM Aβ(1-42) in the presence of increasing concentrations of Cu^2+^, recorded in 200 mM citrate-phosphate buffers adjusted to (**d**) pH 7.0, (**e**) pH 6.0, and (**f**) pH 5.0.

Next, we explored the effect of Cu^2+^ on Aβ(1-42) aggregation at different pHs to mimic the biological conditions that prevail in the endolysosomal pathway, where early accumulation of Aβ aggregates may occur [9]. By successively reducing the pH from 8.0 to 5.0, we also explored the effect of protonation of Cu-coordinating histidines (pK_a_ ~ 6) in the Aβ(1-42) N-terminus. To cover this pH range, a citrate-phosphate buffer was used, which required an increase in the ionic strength to 200 mM compared to the data presented in Figure 2 in order to obtain sufficient buffering capacity at pH 5.0. This in itself decreased the half-time of Aβ(1-42) aggregation from 0.82 ± 0.03 h (20 mM) to 0.20 ± 0.01 h (200 mM) (Figure 3a), consistent with previous observations of salt effects [91,92] and a recent study demonstrating that increased electrostatic screening enhances several mechanistic steps in the self-assembly of Aβ(1-42) [93]. We found that Cu^2+^ slows down Aβ(1-42) aggregation also in 200 mM salt, pH 8.0, (Figure 3b), but the effect is weaker (1.4 times increase in the half-time at 5.2 μM of Cu^2+^ (Figure 3c) compared to a 4.6 times increase at 20 mM of salt (Figure 2a)). This suggests that the catalyzing effects of electrostatic screening on Aβ(1-42) aggregation may to some extent outcompete Cu^2+^-mediated inhibitory actions. 

The effects of pH on the intrinsic aggregation kinetics of Aβ(1-42) are shown in Figure 3a, revealing no or very modest changes above Aβ(1-42)’s isoelectric point (pI = 5.3, [94]), whereas at pH 5.0 the aggregation rate increases, which is also reflected by the change in the aggregation halftimes (0.32 ± 0.05 h in pH 5.0, compared to 0.2 ± 0.01 h at pH 8.0) (Figure 3c). Upon lowering the pH from 8.0 (Figure 3b) to 7.0 or 6.0 (Figure 3d,e), we observed that the Cu^2+^-mediated inhibition became increasingly potent; the aggregation curves display extended lag phases and significantly increased half-times (Figure 3c), particularly at Cu^2+^:Aβ ratios equal to or exceeding 1:1. This behavior could relate to differences in the coordination mode and binding stoichiometry of the Cu^2+^:Aβ complex, which has been shown to be pH-dependent in several studies [64,66,68,69]. Particularly, the binding of a second Cu^2+^ ion appears more prominent in mildly acidic (pH 6.6) conditions [70], which could explain the sudden increase in the degree of inhibition of aggregation that we observe at a Cu^2+^:Aβ ratio of 1:1 and above at pH 7.0 and 6.0, but not at pH 8.0. The lack of effect of Cu^2+^ additions at pH 5.0 (Figure 3f) is most likely due to a loss of binding, related to the full protonation of His residues [67], even if one report suggests a weak Cu^2+^ binding to Aβ monomers at this pH [66]. This result further implies that Aβ(1-42) may release Cu^2+^ during endolysosomal acidification following endocytic uptake [95], and that this in turn could lead to both aggravated aggregation of Aβ and putative toxicity due to free Cu^2+^ ions. 

### 3.3. Cu^2+^-Mediated Inhibition of Aβ(1-42) Aggregation Affects the Fibril Elongation Step

**Figure 4:**
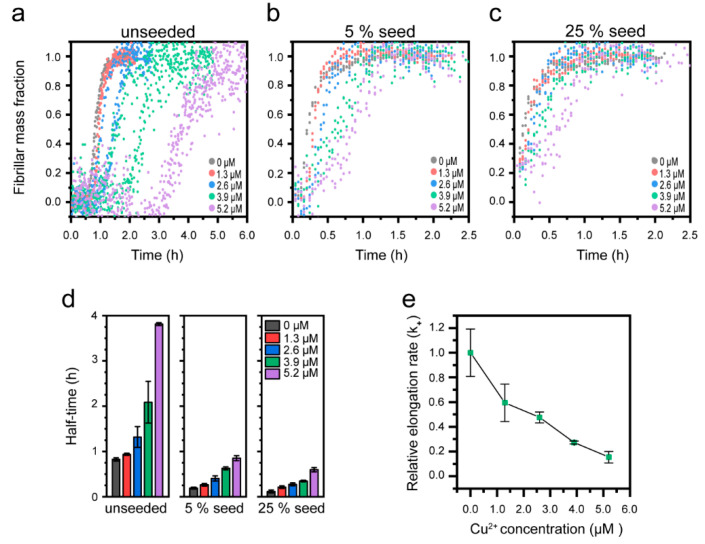
Effect of Cu^2+^ on seeded Aβ(1-42) amyloid formation at pH 8.0 (a–**c**) Normalized kinetic profiles of 2.6 µM Aβ(1-42) in presence of indicated concentrations of Cu^2+^ in reactions with (**a**) no seeds, (**b**) 5% seeds, and (**c**) 25% seeds. (**d**) Half-times of Aβ(1-42) aggregation, extracted from the data in (**a**–**c**). Error bars represent the standard deviation (n = 3). (**e**) Relative elongation rate of 2.6 µM Aβ(1–42) in presence of 25% seeds as a function of Cu^2+^ concentration, as estimated using a linear approximation of the kinetic slope up until a fibril mass fraction of 0.5. All the rate constants are relative to that of 2.6 µM of Aβ(1-42) in absence of Cu^2+^.

It has been shown in several independent studies that the rate-limiting step in unseeded aggregation of Aβ(1-42) is secondary nucleation [14,18,51]. Aggregation modulators can, however, act on different mechanistic steps [96]. To investigate how Cu^2+^ affects the mechanism of Aβ(1-42) aggregation, we returned to pH 8.0 and low ionic strength conditions and explored how the aggregation kinetics of Aβ(1-42) were affected by the addition of pre-formed fibril seeds (prepared in the absence of Cu^2+^). Figure 4a shows the kinetic profiles of 2.6 µM Aβ(1-42) with increasing Cu^2+^ and no added seeds (the normalized data from Figure S2a). Figure 4b,c shows the same experiment in the presence of 5 mol% or 25 mol% Aβ(1-42) fibril seeds (mol% on a monomer basis). It is clear that even at strongly seeded conditions (25%), the aggregation of Aβ(1-42) is reduced in a dose-dependent manner by Cu^2+^. This is further visualized by the half-time dependence on the Cu^2+^ concentration, as shown in Figure 4d and Figure S7. The distinct concentration-dependent increase in half-time with 5% and 25% seeds strongly suggests that Cu^2+^-mediated inhibition is not due to a reduction in the primary nucleation rate, since the addition of seeds bypasses this reaction step [97]. Instead, the retardation of Aβ(1-42) aggregation by Cu^2+^ appears to be associated with inhibition of secondary processes and/or elongation. It has been shown that elongation can be distinguished from secondary nucleation at strongly seeded conditions where fibril elongation dominates over all nucleation processes [17].

Since our data show that a significant effect of Cu^2+^ on the aggregation half-times remains with 25% seeds, we proceeded to estimate the elongation rates by fitting a linear curve to the initial growth phase (up to 50% fibril mass fraction) under strongly (25%) seeded conditions (Figure S8a), as described by Abelein et al. [51]. This analysis revealed a Cu^2+^ concentration-dependent decrease in the relative elongation rate (Figure 4e), supporting the notion that Cu^2+^ inhibition acts on fibril elongation. Similar observations have been made in studies on the effect of Zn^2+^ on the aggregation of Aβ(1-40) [51].

**Figure 5:**
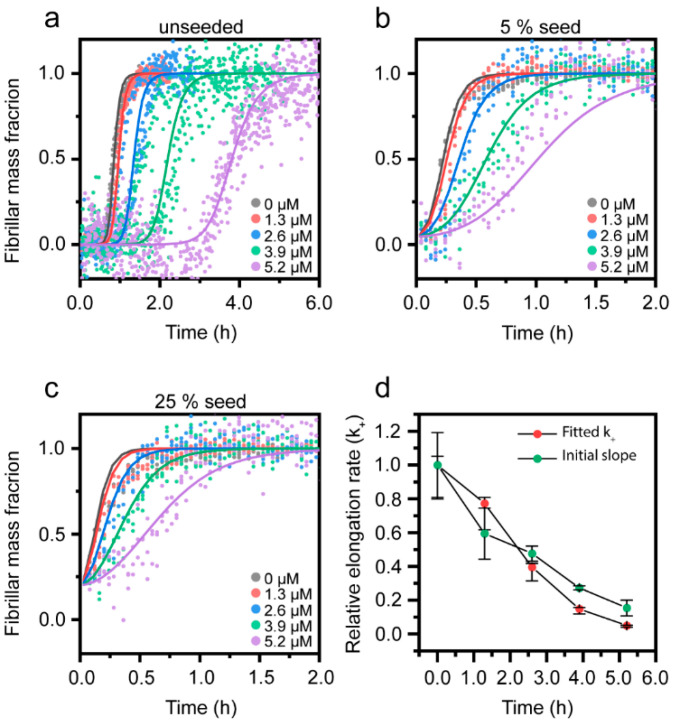
Fitting of the Aβ(1-42) aggregation kinetics in the presence of Cu^2+^ at pH 8.0 to mathematical models of amyloid formation. (**a**–**c**) Normalized kinetic profiles of 2.6 µM Aβ(1-42) at pH 8.0 corresponding to the data in Figure 4. Solid lines are the global fits to data using a model for Cu^2+^-dependent variation in the elongation rate constant (k_+_). (**d**) Relative change in the elongation rate constant (k_+_) determined from the fitting of the data in (**a**–**c**) compared to the relative rate determined via the linear approximation of the initial slope at 25% seeds in Figure 4e. All the rate constants are relative to that of 2.6 µM of Aβ(1-42) in the absence of Cu^2+^.

To quantitatively determine the elongation rate constants together with the rates for other steps in the reaction, we performed a global fitting to the combined data sets of unseeded and seeded ThT emission kinetics in the Amylofit web interface, using a secondary nucleation model for seeded amyloid growth [85]. This allowed us to independently determine the microscopic rate constants for primary nucleation (k_n_), elongation (k_+_), and secondary nucleation (k_2_). The elongation rate constant (k_+_) was probed as a fitting parameter, (allowed to vary with the Cu^2+^ concentration, but kept constant across seeding concentrations), and the two other rate constants were fitted as fixed global parameters. The resulting fits are shown in Figure 5a–c, and the associated rate constants are given in Table 1. We repeated the analysis with, respectively, k_n_ and k_2_ as fitting parameters (Figure S9), further substantiating that Cu^2+^ cannot inhibit the primary nucleation step of the Aβ(1-42) aggregation reaction (Figure S9a–c).

From a mathematical perspective, it is more difficult to distinguish between the fits for secondary nucleation inhibition (k_2_, Figure S9d–f) and elongation inhibition (k_+_, Figure 5a–c), but the latter (which also has a slightly better goodness of fit, 0.0206 vs 0.0214; the corresponding parameter for the rejected k_n_ fit was 0.0265) captures better the initial lagging behavior at high Cu^2+^ ratios. Furthermore, increasing the Cu^2+^ concentration results in longer lag times together with successively decreased steepness of the slopes of the aggregation kinetic curves (Figure 4a), which is expected for elongation inhibitors [16]. The change in the elongation rate constant (k_+_) with increasing Cu^2+^ concentration determined from the fitting (Figure 5e, Table 1) overlaps very well with the relative elongation rate constants estimated using the initial slope of the kinetic profiles (Figure 4e). We therefore conclude that Cu^2+^ inhibits the Aβ(1-42) aggregation by interfering with the fibril elongation step. In order to compare the fitted rate constants for the Aβ(1-42) aggregation in the absence of Cu^2+^ with previously published values, we calculated the combined rate constants k_n_k_+_ and k_2_k_+_ from the data given in Table 1. This yielded 2.5 × 10^7^ M^−2^h^−2^ and 8.6 × 10^18^ M^−3^h^−2^ for k_n_k_+_ and k_2_k_+_, in good agreement with previous reports [14,51], demonstrating the robustness of this Aβ(1-42) aggregation system.

Our results show that Cu^2+^ reduces the elongation rate of Aβ(1-42) by 60% at a 1:1 Cu^2+^:Aβ ratio and by 95% at a 2:1 ratio. These values can be compared to the elongation inhibitory effect of Zn^2+^ on Aβ(1-40), which resulted in an 80% reduction in the rate constant at sub-stoichiometric concentrations (2.5:20 molar ratio) [51] and to the extracellular chaperone clusterin, which elicits the strong inhibition of elongation even at molar ratios as low as 1:100 [80]. In comparison, the Cu^2+-^mediated inhibition appears to be weaker.

Fibril elongation occurs via monomer addition to the growing fibril end, and the inhibition could therefore be envisioned to result from Cu^2+^ binding to the attaching monomer, the growing fibril, or both, especially since Cu^2+^ has been reported to bind both Aβ(1-42) monomers and fibrils with a comparable affinity [63]. Most studies suggest that elongation occurs via templating interactions, where the misfolding rearrangement is aided by monomer contacts to the fibril end [98,99,100]. Molecular dynamics simulations have suggested that it is the N-terminal part of the Aβ peptide that forms the initial contact with the growing fibril end [101]. It can therefore be envisioned that the Cu^2+^ interaction, promoting loop-formation in the N-terminus of either the free Aβ(1-42) monomer or the Aβ(1-42) entities at the fibril end (Figure 1a), imposes conformational restrictions or charge repulsions that impede monomers from attaching to the fibril ends.

### 3.4. Effect of Cu^+^ on Aβ(1-42) Aggregation

**Figure 6:**
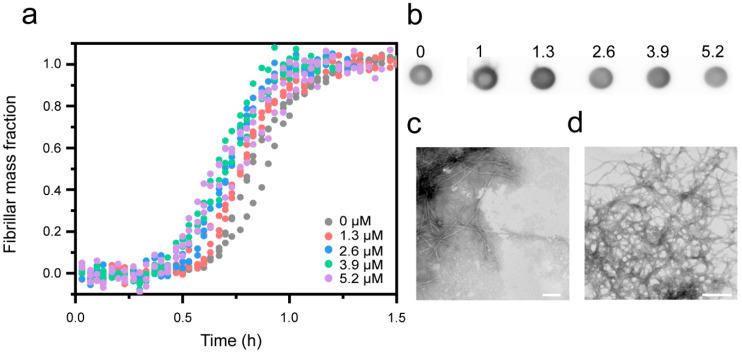
Aβ(1-42) amyloid formation in the presence of Cu^+^ at pH 8.0. (**a**) Normalized kinetic profiles of 2.6 µM Aβ(1-42), measured by ThT (5 µM) fluorescence, in the presence of indicated concentrations of CuCl_2_ and 5x molar excess 1,4-dithiothreitol (DTT) to generate Cu^+^. The non-normalized dataset is shown in Figure S2b. (**b**) Dot blot showing LOC-positive Aβ(1-42) species at the endpoint of the kinetic experiments shown in (**a**). The full, uncropped image of the dot-blot membrane is shown in Figure S4. (**c**,**d**) TEM images of 2.6 µM Aβ(1-42) amyloid fibrils formed in the presence of (**c**) 1.3 µM and (**d**) 5.2 µM Cu^2+^. The scale bars represent 200 nm.

Since copper can exist in, and cycle between, two redox states (Cu^+^ and Cu^2+^), we also studied the effect of Cu^+^ on the aggregation kinetics of Aβ peptides. Notably, this has not been explored previously, in part because Cu^+^ is difficult to work with in aired solutions. To create and keep Cu^+^ in solution, we used the reducing agent dithiothreitol (DTT), in similarity to work with Cu^+^ chaperones [82]. The aggregation of Aβ(1-42) in presence of CuCl_2_ and a 5x molar excess of DTT to Cu (creating Cu^+^) resulted in a modest but reproducible concentration-dependent increase in the aggregation rate of Aβ(1-42) (Figure 6a) (for two additional replicates of the experiments, see supporting Figure S10), thus opposite to the effect of Cu^2+^. Control experiments showed that DTT on its own had no effect (Figure S11). The kinetic results were confirmed by dot blot (Figure 6b, Figure S4), showing no decrease in the fibril yield in the presence of Cu^+^. TEM imaging revealed the presence of typical amyloid fibril aggregates at both sub- and super stochiometric Cu^+^:Aβ ratios (Figure 6c,d), similar to those observed with Cu^2+^, suggesting that copper ions and redox state have no major effect on the morphology of formed amyloid fibrils.

### 3.5. Cu^+^ Enhances Aβ(1-42) Aggregation by Catalysis of Primary Nucleation

Cu^+^ has been reported to bind Aβ(1-42) via bidentate coordination to imidazoles of His13 and His14, located adjacent to the N-terminal side of the first β-sheet in the fibril core, but His6 may also be involved (Figure 1d, [65]). In order to rationalize how this leads to the enhancement of Aβ(1-42) aggregation, we performed seeded experiments analogous to those explained in Section 3.3.

**Figure 7:**
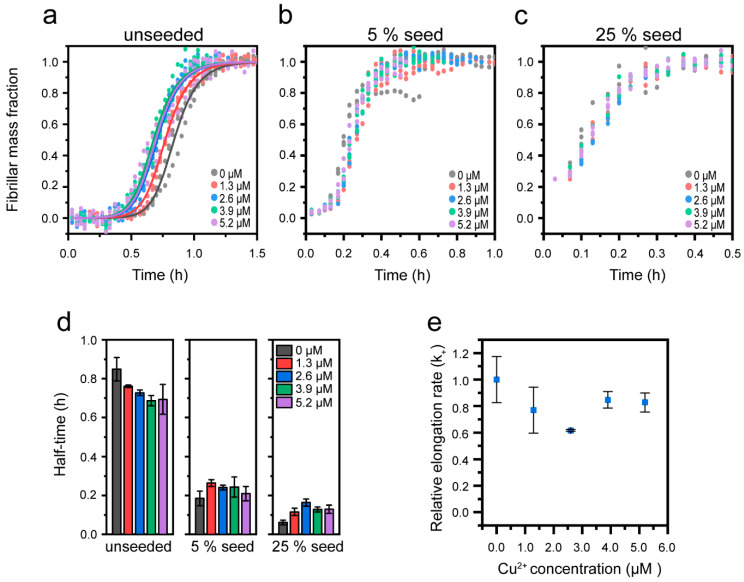
Effect of Cu^+^ on seeded Aβ(1-42) amyloid formation at pH 8.0. (**a**–**c**) Normalized kinetic profiles of 2.6 µM Aβ(1-42) in presence of indicated concentrations of Cu^2+^ in reactions with (**a**) no seeds, (**b**) 5% seeds, and (**c**) 25% seeds. (**d**) Half-times of Aβ(1-42) aggregation extracted from the data in (**a**–**c**). Error bars represent the standard deviation (n = 3). (**e**) Relative elongation rate of 2.6 µM Aβ(1-42) in the presence of 25% seeds as a function of Cu^2+^ concentration, as estimated using a linear approximation of the kinetic slope up until a fibril mass fraction of 0.5. All the rate constants are relative to that of 2.6 µM Aβ(1-42) in the absence of Cu^2+^. The solid lines in (**a**) are the global fits to data using a model for Cu^2+^-dependent variation in the combined rate constant for primary nucleation and elongation (k_n_k_+_).

We find that whilst Cu^+^ shortens the half-time of unseeded Aβ(1-42) aggregation from 0.84 ± 0.06 to 0.69 ± 0.07 (Figure 7a,d), there is no significant effect of Cu^+^ on seeded reactions (Figure 7b–d), suggesting that secondary pathways are not affected. Consistently, Cu^+^ had no effect on the initial slope of the kinetic traces under strongly seeded (25%) conditions, and therefore not on the relative elongation rate constants (Figure 7e and Figure S7b). Furthermore, the observed characteristic shortening of the lag time, without apparent alterations to the steepness of the slope of the aggregation curves [97], strongly supports that the Cu^+^-mediated catalysis of the Aβ aggregation acts on primary nucleation. Although in aired solutions, Cu^+^ will over time oxidize to Cu^2+^, it has been previously shown that under reducing conditions (1-fold excess ascorbate) Cu^+^ remains stable for at least 1.5 h [65], which is longer than the experimental time span in Figure 6a.

We globally fitted the unseeded Aβ(1-42) aggregation data in the presence of Cu^+^ using the Amylofit web interface [85] and a secondary nucleation model for unseeded amyloid growth. The fit (solid lines in Figure 7a) shows that the mild aggregation-enhancing effect of Cu^+^ is well-described by an increase in the combined primary nucleation and elongation rate constant k_n_k_+_; the resulting combined rate constants from the fitting are shown in Table 2. 

There are relatively few studies investigating the Cu^+^–Aβ interaction, but due to the preferred linear coordination geometry involving His13 and His14 [65,102], it is conceivable that Cu^+^ cannot simultaneously bind these two ligands in the fibrillar state (due to steric restrictions imposed by the alternating directions of side-chain residues in or near the cross-β core (Figure 1b)). However, it is plausible that Cu^+^ may interact with His13 and His14 residues on different monomers in the amyloid fibrils. Our data would then be consistent with a model in which Cu^+^ catalyzes the formation of Aβ(1-42) dimers, which are the minimal elongation-competent Aβ(1-42) units [14], thus promoting primary nucleation.

## 4. Conclusions

**Figure 8:**
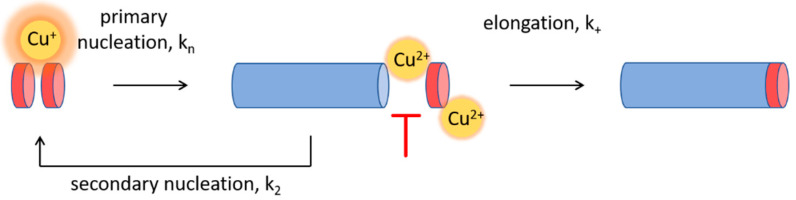
Schematic model showing the differential effects of Cu^2+^ and Cu^+^ on the amyloid formation of Aβ(1-42), with Cu^2+^ acting in an inhibitory manner on elongation and Cu^+^ mildly affecting primary nucleation.

This study explores the effect of copper ions on Aβ(1-42) amyloid formation in vitro, identifying effects on the overall aggregation rate as well as on discrete mechanistic steps of the aggregation pathway (illustrated in Figure 8). Our results extend the growing body of literature, suggesting that Cu^2+^, the major extracellular copper form [21], acts in an inhibitory manner on fibril formation [44,45,46,47,48,49,50]. We solidify this view by seeding experiments and kinetic analyses, which conclusively show how Cu^2+^ primarily impedes fibril elongation at both sub- and super-stoichiometric ratios and in a concentration range that is relevant to the conditions that prevail in the brain. The Cu^2+^-mediated inhibition is modest in comparison to other elongation inhibitors, including Zn^2+^ (see above and [51]). This is consistent with observations of the significant accumulation of Cu^2+^ in AD plaques [21], and suggests that Cu^2+^ impedes, rather than completely blocks, Aβ(1-42) aggregation. Although the inhibition of Aβ(1-42) aggregation is a tractable goal to limit the onset and progression of AD, it is important to recognize that elongation inhibition, under conditions where primary and secondary nucleation proceeds, can instead drive the formation of small fibrils and oligomers [12,16]. It is therefore possible that Cu^2+^, despite its ability to reduce the amount of mature fibrils formed, as we report here, contributes to Aβ toxicity in the brain. Furthermore, although we describe a strong stoichiometry-dependent inhibitory effect of Cu^2+^ at a mildly acidic pH, we also report a loss of function at pH 5.0, a condition that prevails in late endosomes and lysosomes and which may drive Aβ aggregation in intraneuronal locations of the brain. 

Furthermore, we demonstrate that the effect of Cu is dependent on the redox state by including the first experimental evidence that Cu^+^ also has an Aβ(1-42) aggregation-modulatory effect. Through kinetic analysis and seeded experiments, we report that Cu^+^ accelerates fibril formation by mild catalysis of primary nucleation, possibly related to Cu^+^-mediated dimer formation. This finding is important, particularly in relation to observations that the redox cycling of Aβ-bound Cu can contribute to the generation of reactive oxygen species in the brain [32]. Our results suggest that such reactions, which result in Cu^+^ bound to Aβ, may also accelerate aggregation, thereby constituting a dual toxic mechanism. Altogether, our study sheds more light on the possible pathological relevance of Cu in Alzheimer’s disease and suggests a complex pivotal role of Cu in Aβ(1-42) aggregation modulation that warrants further investigation in biological in vivo environments. The therapeutic value of Cu–Aβ(1-42) interactions should also be taken into account in future strategies to develop disease-modifying treatments.

## Figures and Tables

**Table 1:** Rate constants for Aβ(1-42) aggregation in the presence of Cu^2+^. Rate constants were determined by fitting the seeded kinetic data with k_+_ as a dependent variable and k_n_ and k_2_ as globally fitted fixed parameters (e.g., elongation inhibition). The fitted values for k_n_ and k_2_ were $1.2\times 10^{{-1}_{-9.2\times 10^{-3}}^{+1.9\times 10^{-2}}}M^{-1}h^{-1}$ and $4.1\times 10^{10_{-3.5\times 10^{9}}^{+6.7\times 10^{9}}}M^{-2}h^{-1}$ respectively. The upper/lower errors define the interval in which the rate constant variation does not significantly changing the goodness of fit [85].

[Cu^2+^]	k_+_	Upper/Lower Error
(µM)	(M^−1^h^−1^)	(M^−1^h^−1^)
0	2.1 × 10^8^	+1.1 × 10^7^
		−4.2 × 10^7^
1.3	1.6 × 10^6^	+7.5 × 10^6^
		−3.2 × 10^7^
2.6	8.2 × 10^7^	+4.5 × 10^6^
		−1.7 × 10^7^
3.9	3.1 × 10^7^	+1.6 × 10^6^
		−6.2 × 10^6^
5.2	1.0 × 10^7^	+5.3 × 10^5^
		−2.1 × 10^6^

**Table 2:** Rate constants for Aβ(1-42) aggregation in presence of Cu^+^. Rate constants were determined by fitting unseeded kinetic data and setting the combined rate constant k_n_k_+_ as the dependent variable and the combined rate constant k_2_k_+_ as a globally fitted fixed parameter (e.g., primary nucleation catalysis). The fitted value for k_+_k_2_ was $4.9\times 10^{18_{-1.1\times 10^{17}}^{+6.8\times 10^{16}}}M^{-3}h^{-2}$. The upper/lower errors define the interval in which the rate constant variation does not significantly change the goodness of fit [85].

[Cu^+^]	k_+_k_n_	Upper/Lower Error
(µM)	(M^−2^h^−2^)	(M^−2^h^−2^)
0	5.5 × 10^8^	+5.6 × 10^7^
		−3.9 × 10^7^
1.3	1.4 × 10^9^	+1.3 × 10^8^
		−1.0 × 10^8^
2.6	3.2 × 10^9^	+2.6 × 10^8^
		−2.1 × 10^8^
3.9	4.7 × 10^9^	+4.3 × 10^8^
		−2.8 × 10^8^
5.2	3.7 × 10^9^	+3.0 × 10^8^
		−2.4 × 10^8^

## Supplementary Materials

The following supplementary results are available online at https://www.mdpi.com/2218-273X/10/6/924/s1; Figure S1: Size-exclusion (SEC) purification of Aβ(1-42) monomers; Figure S2: Reproducibility of Aβ(1-42) aggregation kinetics; Figure S3: Aβ(1-42) amyloid formation in presence of MgCl_2_; Figure S4: Dot blot; Figure S5: Relative ratio of fibrillar content at different Cu concentrations; Figure S6: Quenching of ThT by Cu^2+^; Figure S7: Half-time of Aβ(1-42) aggregation in presence of Cu^2+^; Figure S8: Initial slope analysis of 25% seeded aggregation curves; Figure S9: Fitting of Aβ(1-42) amyloid kinetic data in presence of Cu^2+^ to models of primary nucleation and secondary nucleation inhibition; Figure S10: Amyloid formation in presence of Cu^+^- extra data; Figure S11: Dithiotreitol (DTT) has no effect on Aβ(1-42) amyloid formation; Table S1: Buffer specifications.
