# Supplementary material for: Redox-Dependent Copper Ion Modulation of Amyloid-β (1-42) Aggregation In Vitro
Source: Biomolecules. 2020 Jun 18;10(6):924. doi: 10.3390/biom10060924 (PMC7355640; doi:10.3390/biom10060924)
Supplement: Supplementary file 1 [file biomolecules-10-00924-s001.pdf]

Supplementary information

Redox-dependent copper ion modulation of amyloid-β (1-42) aggregation *in vitro*

Nima Sasanian [1], David Bernson [1], Istvan Horvath[1], Pernilla Wittung-Stafshede[1] and Elin K. Esbjörner[1,*]

[1] Department of Biology and Biological Engineering, Chalmers University of Technology, 412 96 Gothenburg, Sweden. sasanian@chalmers.se; david.bernson@chalmers.se; istvanh@chalmers.se; pernilla.wittung@chalmers.se;

* Correspondence: eline@chalmers.se; Tel.: +46 31 772 51 20

1. Supporting figures

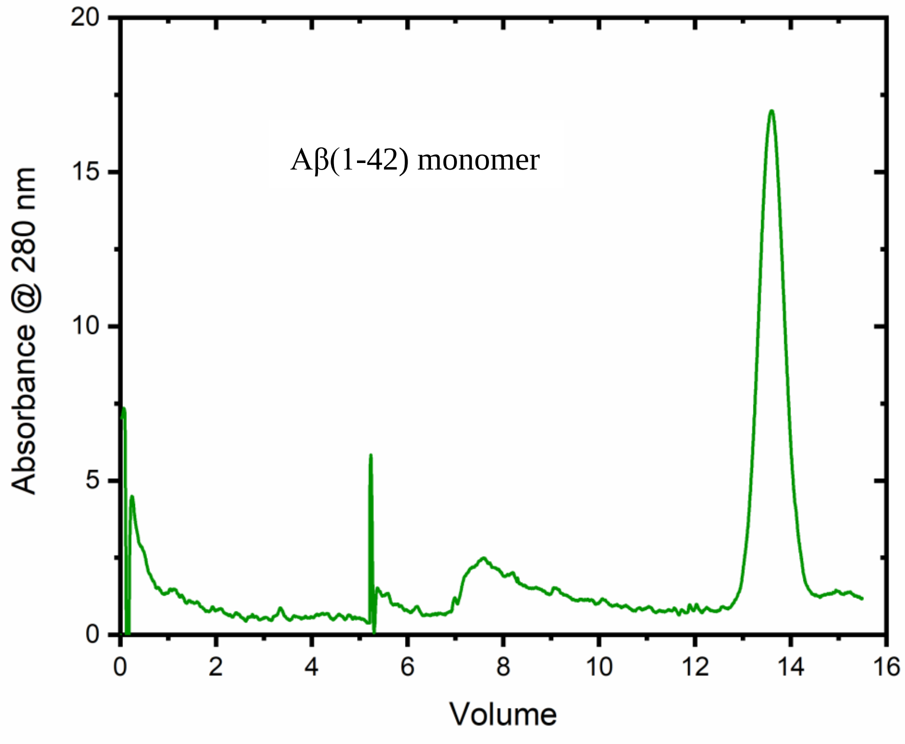

Figure S1. Size-exclusion (SEC) purification of Aβ(1-42) monomers. Chromatogram showing (SEC) preparation of monomeric Aβ(1-42), eluted as one single peak from Superdex 75 10/300 column.

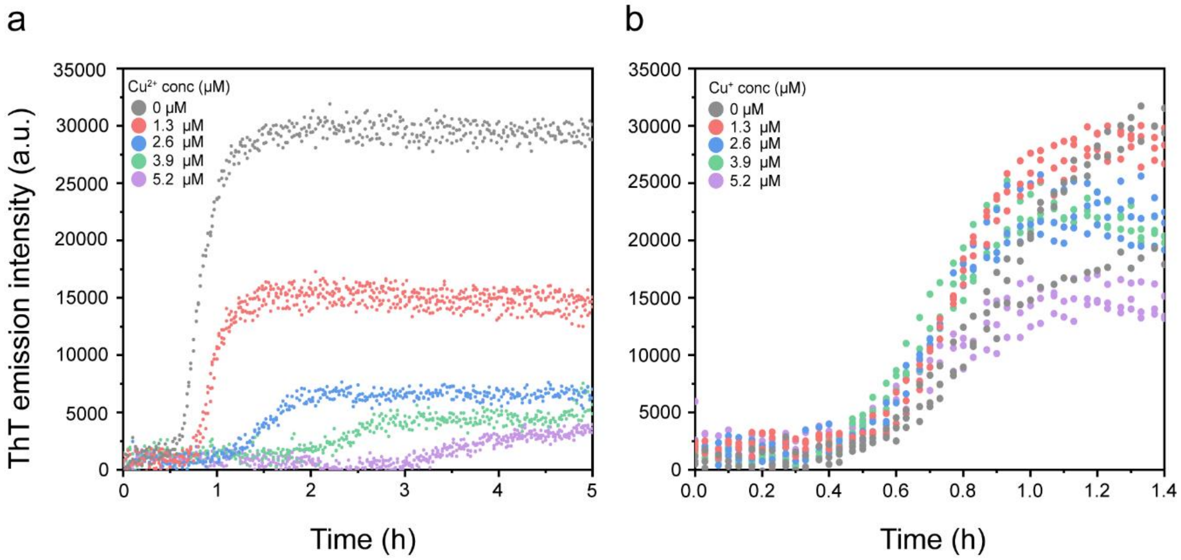

Figure S2. Reproducibility of Aβ(1-42) aggregation kinetics. (a) Replicates (n=3, curves overlaid) showing the reproducibility of the kinetic curves shown in Figure 2a of the main text. The Aβ(1-42) concentration was 2.6 μM Aβ(1-42) and the ThT concentration (5 μM). The figure legend indicates the concentration of $CuCl_2$. **(b)** Replicates (n=3, curves overlaid) showing the reproducibility of the kinetic curves shown in Figure 6a of the main text. The Aβ(1-42) concentration was 2.6 μM Aβ(1-42) and the ThT concentration (5 μM). The figure legend indicates the concentration of $CuCl_2$:DDT (1:5).

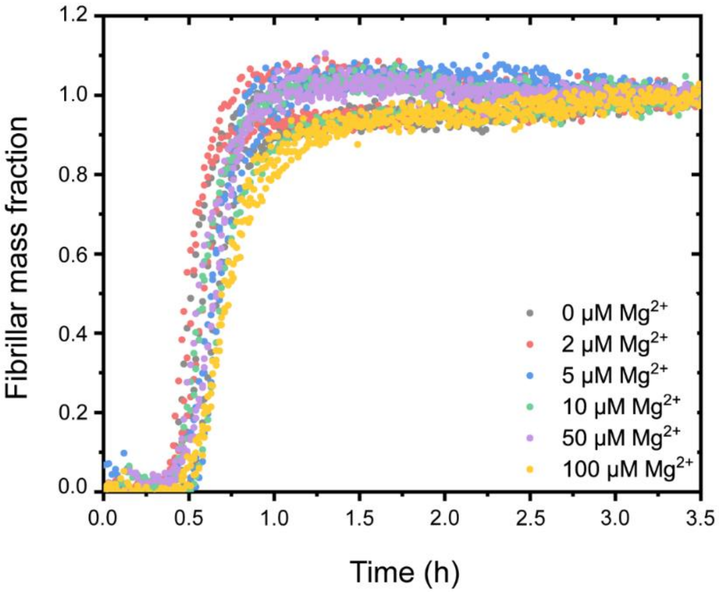

Figure S3. Aβ(1-42) amyloid formation in presence of $MgCl_2$. Amyloid formation kinetics of 3.3 μM Aβ(1-42) in presence of increasing concentrations of $MgCl_2$, monitored by the change of fluorescence emission of 5 μM ThT. Triplicate measurements overlaid.

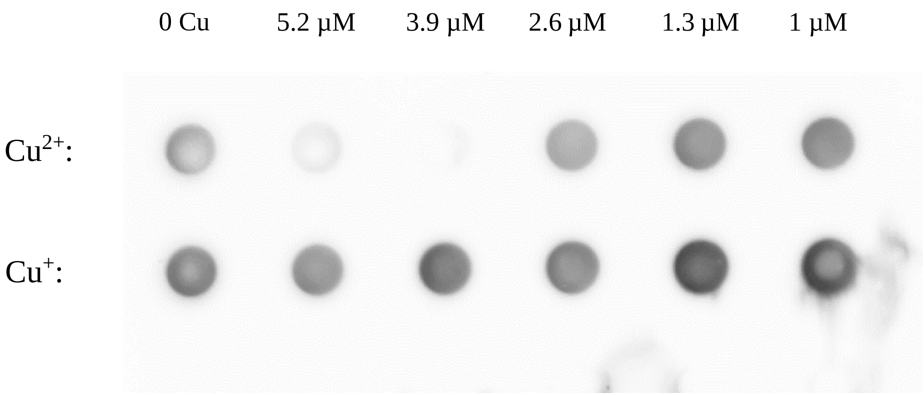

Figure S4. Dot blot. Full uncropped imaged of dot blot showing LOC positive Aβ(1-42) species at the endpoint of the kinetic experiments shown in Figures 2a and 6a of the main text.

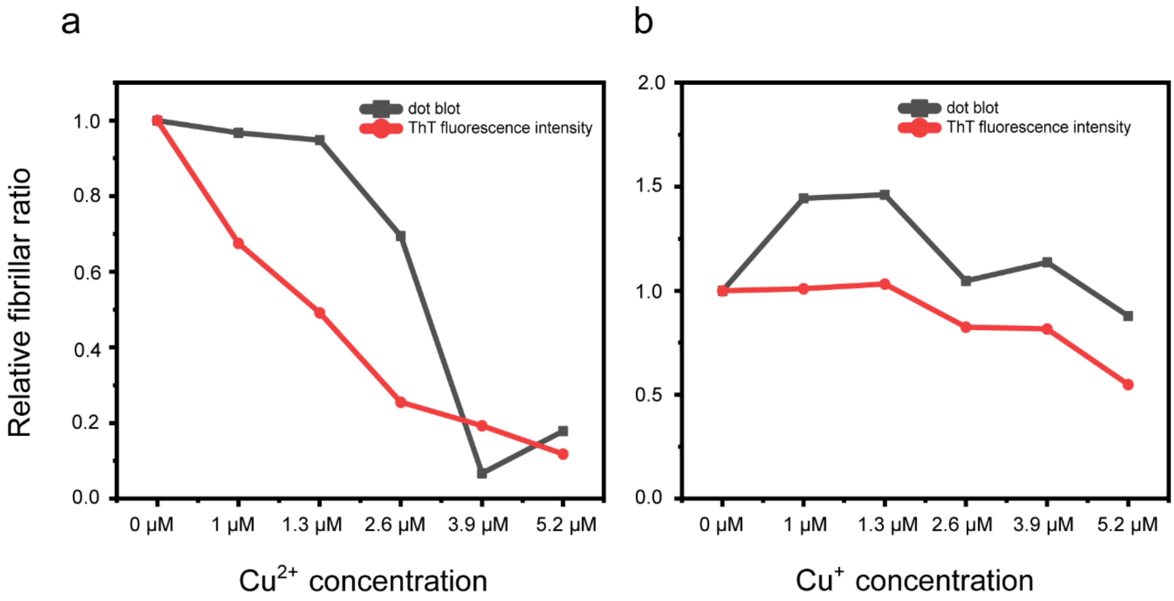

Figure S5. Relative ratio of fibrillar content at different Cu concentrations. Fibril yield (relative to 0 µM reaction) determined by densiometric analysis of the dot blot in Figure S4 **(black)** and by change in end-point ThT fluorescence of the data in Figure 1 and Figure 6 in the main text **(red). (a)** Effect of Cu^{2+} **(a)** and **(b)** effect of Cu^{+}.

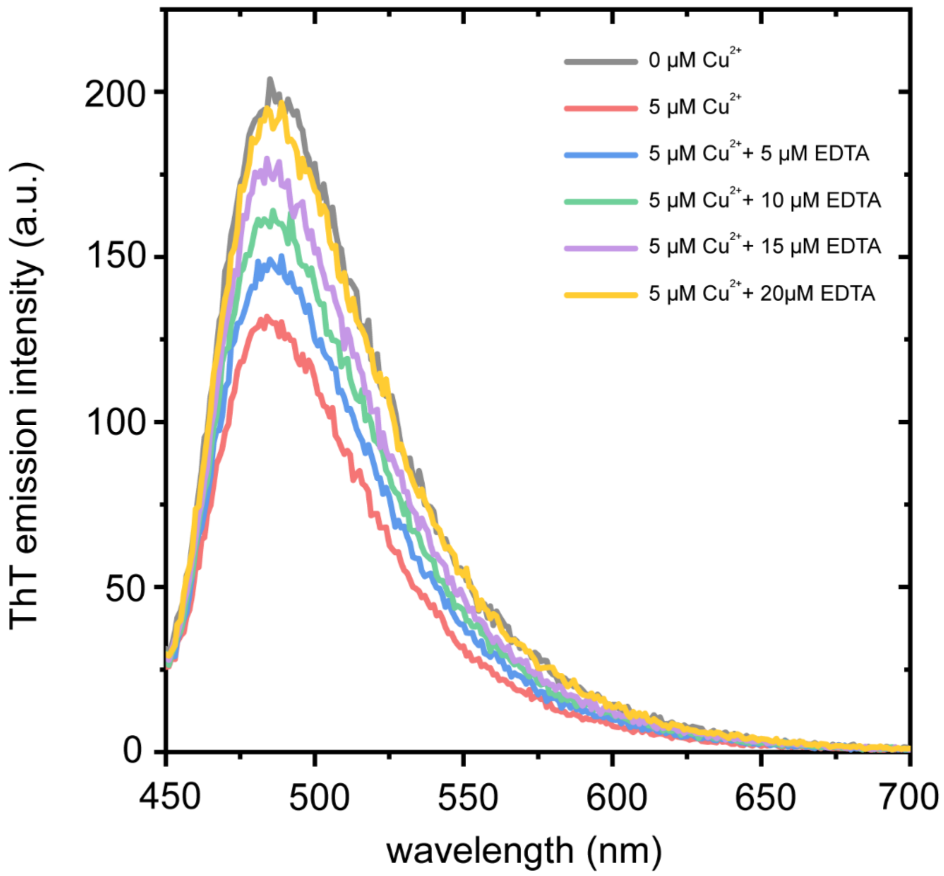

Figure S6. Quenching of ThT by Cu^{2+}. ThT fluorescence spectra recorded in presence of 5 µM Aβ(1-42) amyloid fibrils with indicated concentrations of Cu^{2+} and EDTA. All samples contain 10 µM ThT. The addition of Cu^{2+} results in quenching, reversible by the subsequent titration with EDTA. Steady-state ThT fluorescence emission spectra were recorded in 70 µL with 1 cm path length, quartz micro-cuvette (Hellma Analytics) using a Varian Eclipse fluorimeter (Agilent Technologies). ThT emission spectra between 450 and 700 nm were recorded upon excitation at 440 ± 5 nm. Emitted light were collected through 10 nm monochromator by setting photomultiplier gain to 700 V.

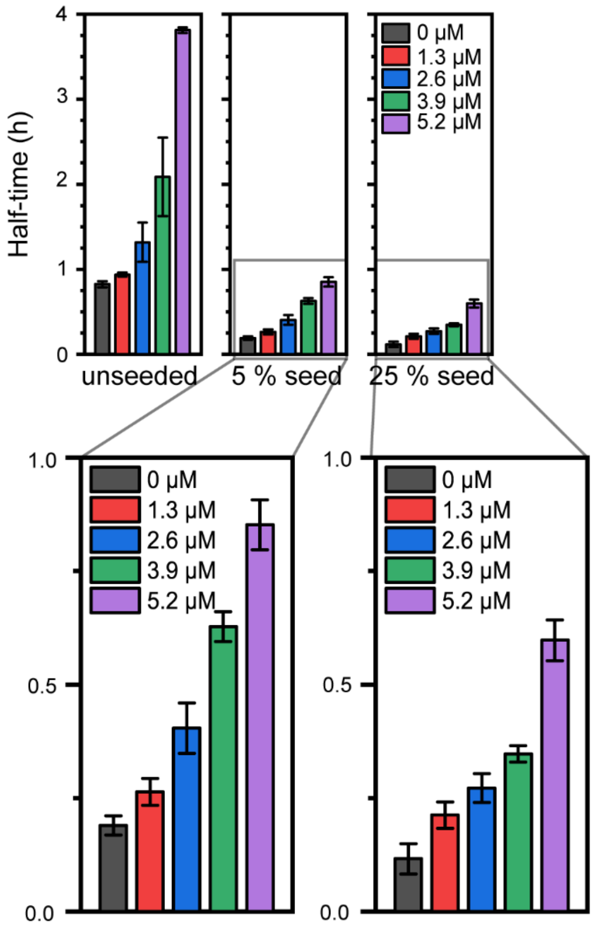

Figure S7. Magnification of aggregation half-times of seeded Aβ(1-42) aggregation in presence of Cu^{2+}, corresponding to the data shown in Figure 4d.

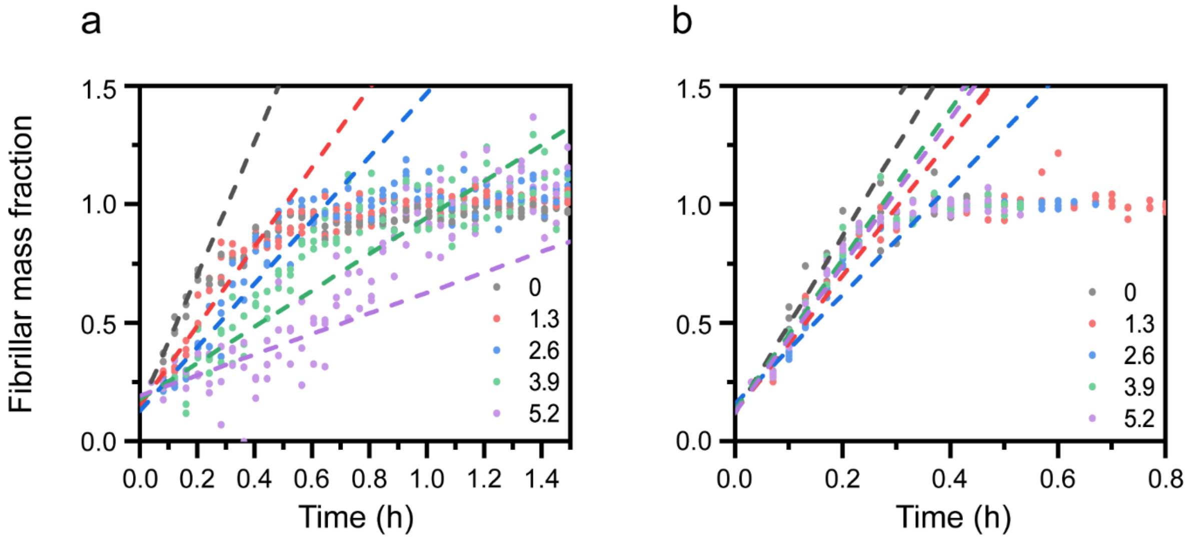

Figure S8. Initial slope analysis of 25% seeded aggregation curves to estimate the relative elongation rate. (a) Normalised kinetic profiles of amyloid formation of 2.6 μM Aβ(1-42) in presence of 25% (mol/mol) fibril seeds and indicated amounts of Cu^{2+}. Dashed lines represent linear fits to data before the fibril mass fraction reaches 0.5. **(b)** Normalised kinetic profiles of amyloid formation of 2.6 μM Aβ(1-42) in presence of 25% (mol/mol) fibril seeds and indicated amounts of Cu^{+}. Dashed lines represent linear fits to data before the fibril mass fraction reaches 0.5.

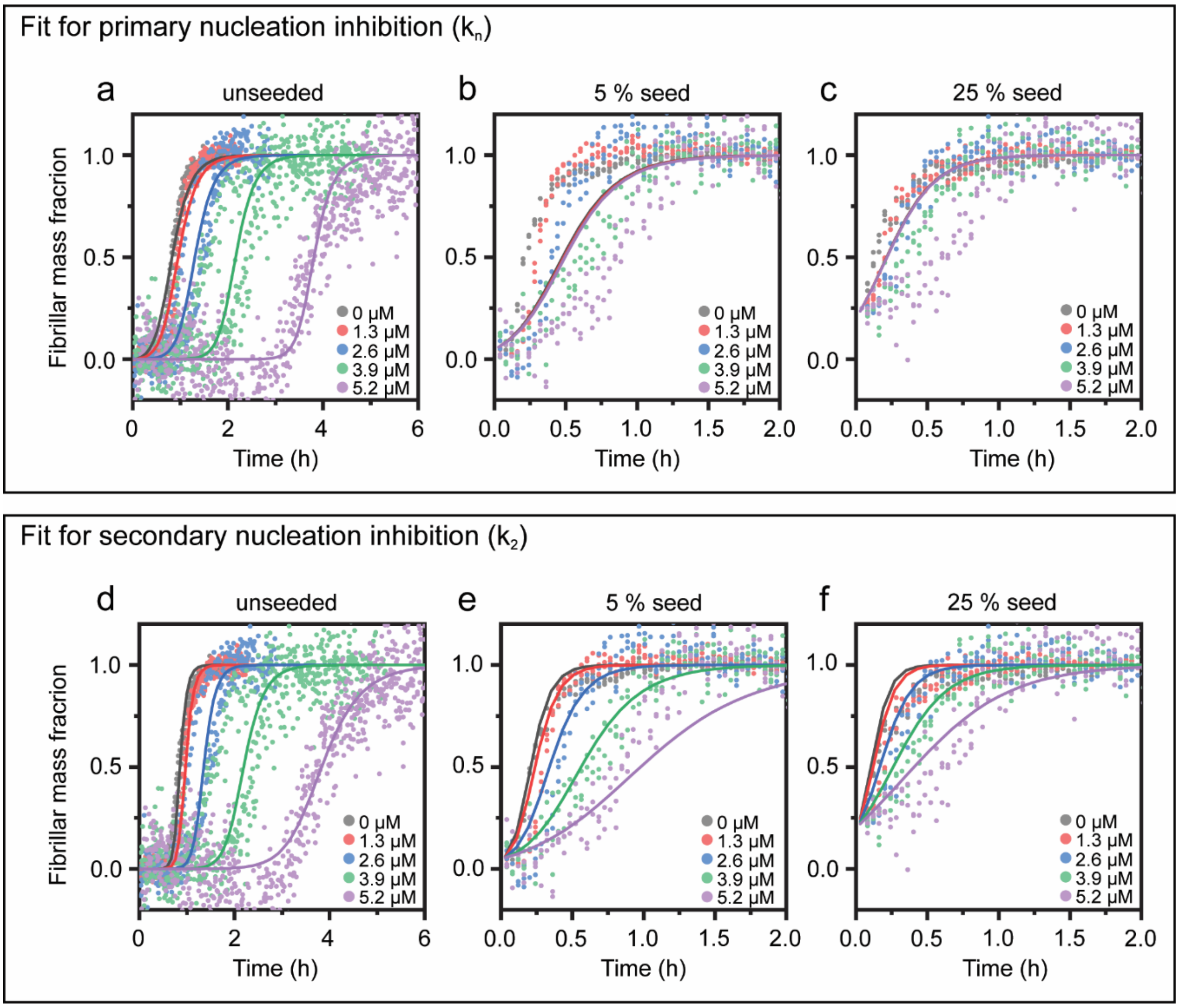

Figure S9. Fitting of Aβ(1-42) amyloid kinetic data in presence of Cu^{2+} to models of primary nucleation and secondary nucleation inhibition. Normalised kinetic profiles of 2.6 μM Aβ(1-42) aggregating in presence of indicated concentrations of $CuCl_2$ and increasing concentration of preformed fibril seeds. Solid lines are predictions based on global kinetic modelling of **(a-c)** reduction of the primary nucleation rate constant (k_n), and **(d-f)** reduction of the secondary nucleation rate constant (k_2).

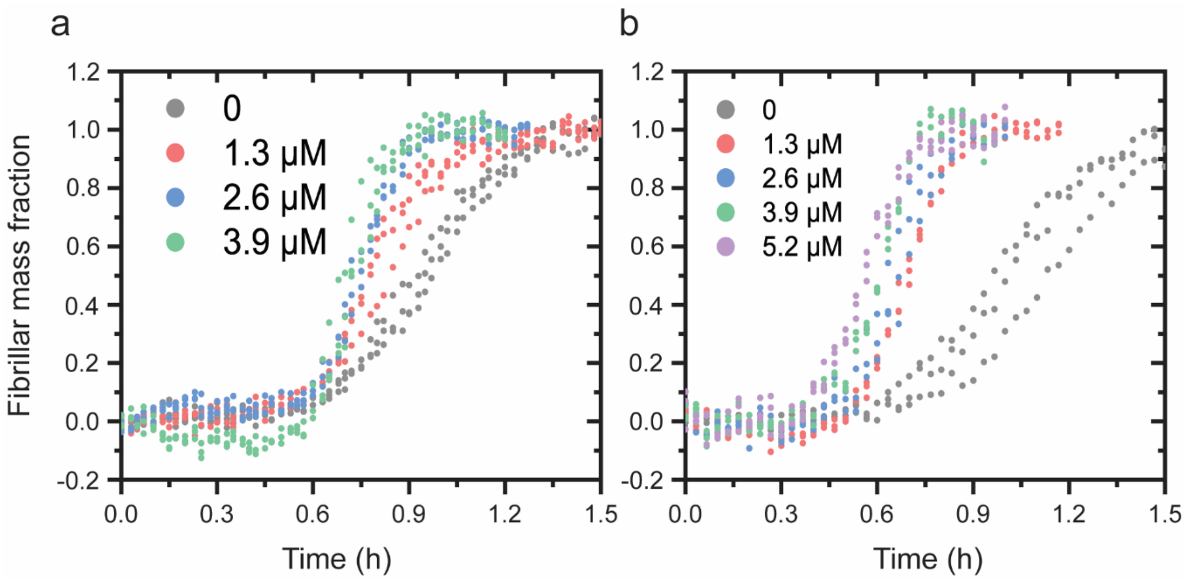

Figure S10. Additional data sets showing the effect of Cu⁺ on the aggregation rate of Aβ(1-42). Each figure shows individual experiments performed on different days, each condition is assayed in triplicate (n=3), all kinetick traces are overlaied. in triplicate (n=3). The data is presented as normalized kinetic curves. The experimental conditions were the same as for the data shown in Fig. 6a in the main text.

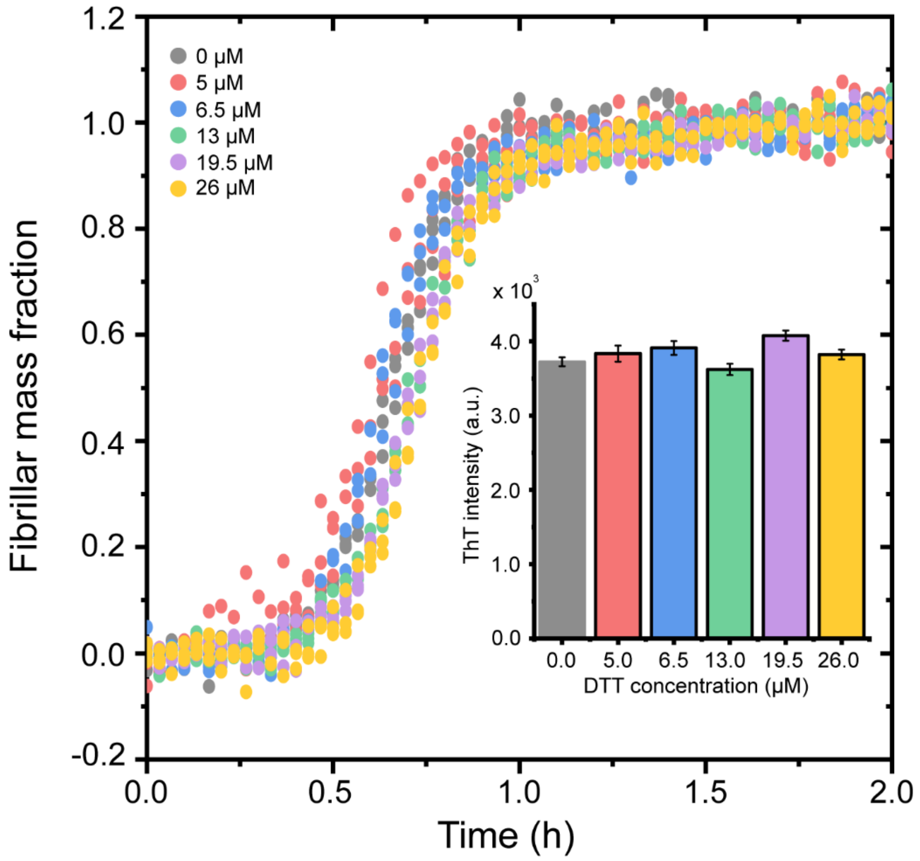

Figure S11. Dithiotreitol (DTT) has no effect on Aβ(1-42) amyloid formation. Normalized kinetic profiles of 2.6 μM Aβ(1-42) in presence of indicated concentrations of ditiotreitol (DTT) monitored by the change of fluorescence emission of 5 μM ThT. **Insert:** Endpoint ThT fluorescence intensity of 2.6 μM Aβ(1-42) in presence of indicated concentrations of DTT.

2. Supporting tables

Table S1. Buffer specifications

pH	buffer	acidic solution	basic solution	acidic solution concentration [mM]	basic solution concentration [mM]
8	phosphate (20 mM)	monobasic sodium phosphate	dibasic sodium phosphate	1.06	18.94
8	Phosphate (200 mM)	monobasic sodium phosphate	dibasic sodium phosphate	10.6	189.4
7	Phosphate (200 mM)	monobasic sodium phosphate	dibasic sodium phosphate	78	122
6	Phosphate (200 mM)	monobasic sodium phosphate	dibasic sodium phosphate	175.4	24.6
5	citrate-phosphate (200 mM)	citric acid	dibasic sodium phosphate	48.6	102.8
